# Primer registro de accidentes ofídicos por mordedura de *Micrurus ortoni* y *Micrurus hemprichii* (Serpentes: Elapidae) en Colombia y Perú

**DOI:** 10.7705/biomedica.6112

**Published:** 2021-12-15

**Authors:** Santiago Ayerbe-González, Gloria Esperanza Condiza-Benavides, María José Sevilla-Sánchez

**Affiliations:** 1 Grupo de Investigaciones Herpetológicas y Toxinológicas, Universidad del Cauca, Popayán, Colombia Universidad del Cauca Grupo de Investigaciones Herpetológicas y Toxinológicas Universidad del Cauca Popayán Colombia; 2 Departamento de Medicina Interna, Clínica Los Andes, Tunja, Colombia Departamento de Medicina Interna Clínica Los Andes Tunja Colombia; 3 Grupo de Nutrición, Facultad de Salud, Universidad del Valle, Cali, Colombia Universidad del Valle Grupo de Nutrición, Facultad de Salud Universidad del Valle Cali Colombia

**Keywords:** serpientes de coral, mordeduras de serpientes, Colombia, Perú, Coral snakes, snake bites, Colombia, Perú

## Abstract

Se reportan dos casos de mordedura por serpientes de la especie *Micrurus ortoni* en Colombia y uno por *M. hemprichii* en Perú. En dos de los casos se observó afección neurológica motora leve a moderada y, en todos, se presentó un acentuado trastorno sensitivo con hiperestesia e hiperalgesia irradiada desde el sitio de la mordedura hacia todo el hemicuerpo comprometido. El único paciente que recibió antiveneno, el cual no era específico para el tipo de envenenamiento, desarrolló una reacción al suero equino a los ocho días de su aplicación.

Se presentan y discuten los resultados de las pruebas de laboratorio, incluido el estudio electromiográfico, así como el registro fotográfico de las manifestaciones clínicas y de los agentes causales.

## Abstract (en)

We report two snakebites by *Micrurus ortoni* in Colombia and one by *M. hemprichii* in Perú. In two of the cases, we observed mild to moderate motor neurological involvement and in all patients, there was a marked sensory effect with hyperesthesia and hyperalgesia radiating from the bite site to the entire ipsilateral hemibody. The only patient who received antivenom, which did not correspond with the type of envenomation, developed equine serum sickness eight days after its administration.

The results of the laboratory tests, including an electromyographic study, the photographic record of the clinical manifestations, and the causative agents are presented and discussed.

Las serpientes conocidas comúnmente como corales pertenecen a la familia Elapidae, de la cual se reconocen 30 especies del género *Micrurus* en Colombia y 19 en Perú, y una sola especie del género *Hydrophis* (*Hydrophis platurus*) en los dos países ^1^. Estas serpientes tienen dentición proteroglifa caracterizada por la presencia de un par de colmillos en la maxila, muy pequeños, inmóviles, curvados hacia atrás, y dotados de un conducto semicerrado por donde fluye el veneno ^2^. En Colombia, la gran mayoría de los accidentes elapídicos son ocasionados por *Micrurus mipartitus* y *M. dumerilii,* aunque también se han reportado algunos casos causados por *M. obscurus* y *M. surinamensis*^3^^-^^5^*,* en tanto que, en Perú, no ha habido reportes de casos de accidentes micrúricos hasta el momento ^6^^,^^7^*.*

Las corales verdaderas y las falsas tienen un patrón de colores que combina el rojo (naranja), el amarillo (blanco) y el negro. Los anillos negros siguen un patrón bicolor (*M. mipartitus*), monadal (*M. dumerilii*) o en tríada (*M. ortoni*) ^8^^,^^9^. El ofidismo micrúrico en Colombia es poco frecuente; en algunos estudios se ha reportado una prevalencia de menos del 1 al 3,4 % ^5^^,^^6^. Esto se debe a distintos factores, como sus hábitos secretorios y fosoriales, ya que se adaptan a la vida subterránea y en excavaciones, su comportamiento no agresivo, sus colmillos diminutos y la poca cantidad de veneno inyectable ^3^^-^^5^^,^^9^. 

En este estudio, se presenta el primer reporte de casos de accidente ofídico micrúrico por *M. ortoni* en Colombia y el primero por *M. hemprichii* en Perú. Estas especies se hallan en el piedemonte oriental de la Cordillera Oriental de los Andes y en la Amazonía de Colombia, Venezuela, Ecuador, Brasil, Perú y Bolivia ( figuras 1 y 2).

**Figura 1:**
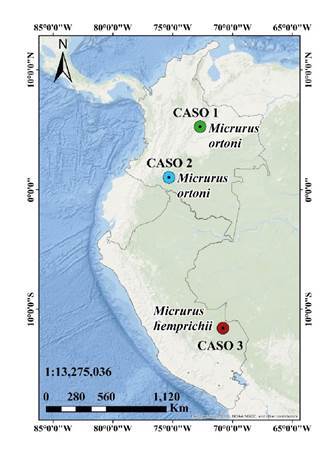
Primeros registros de accidente ofídico causado por las especies *Micrurus ortoni* en Colombia (caso 1 y caso 2) y *Micrurus hemprichii* en Perú (caso 3)

**Figura 2:**
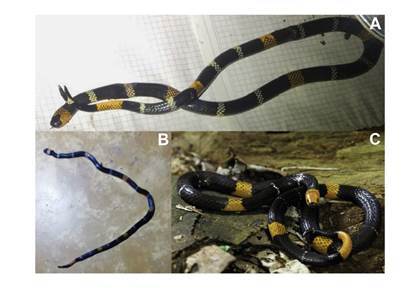
Especies involucradas en los accidentes ofídicos reportados en Colombia y Perú. **A.** *Micrurus ortoni*. Pajarito, Boyacá (Colombia), **B.** *Micrurus ortoni*. Cartagena del Chairá, Caquetá (Colombia), **C.** *Micrurus hemprichii*. Estación Biológica Los Amigos, Madre de Dios (Perú)

Fotografías: Juan Carlos Suárez-Porres (A), Fiorella Escobar-Holguín (B) y Timothy D. Pyne (C)

## Caso 1

En este caso el agente causal fue un individuo de la especie *M. ortoni* (Schmidt 1953), la cual se conoce como “coral lombricera occidental” ^5^^,^^10^, identificado con base en el registro fotográfico (figura 2A). Infortunadamente, el ejemplar fue incinerado por creencias religiosas.

Se trata de una mujer de 24 años, que pisó accidentalmente la serpiente y fue mordida en la cara interna de la región talar del miembro inferior derecho a las 18:15 horas del 11 de septiembre de 2020, encontrándose en la parte posterior de su casa, situada en el casco urbano de Pajarito, noreste de Boyacá, a 1.400 msnm, en el piedemonte oriental de la Cordillera Oriental de los Andes.

La paciente llegó al centro de nivel I de atención (centro de salud básico), a 30 minutos del sitio del accidente ofídico, refiriendo dolor intenso en la extremidad afectada, irradiado a la región lumbar, así como sensación de parestesias generalizadas y cefalea intensa. El acompañante enseñó la fotografía al médico, quien determinó que no se trataba de una serpiente venenosa y canalizó una vena periférica.

En el examen físico, su tensión arterial era de 100/51 mm Hg, la frecuencia cardíaca de 115 por minuto, la frecuencia respiratoria de 17 por minuto, la saturación de oxígeno de 89 % y la temperatura de 36,0 ^º^C; su peso era de 64 kg, la estatura de 163 cm y el índice de masa corporal (IMC) de 24 kg/m^2^.

**Figura 3:**
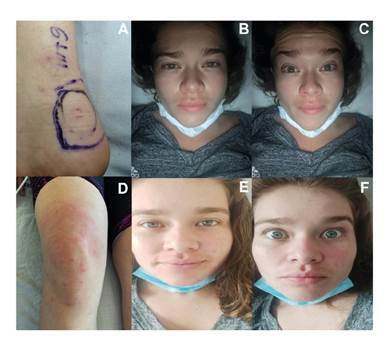
Ofidismo micrúrico por M. ortoni (Pajarito). A. Área de la mordedura. B. Facies de Rosenfeld. C. Paresia del III par craneano. D. Enfermedad del suero (séptimo día). E. Facies normal (octavo día). F. Eritema facial (octavo día)

Se encontraba alerta, agitada y ansiosa, con pupilas isocóricas y fotorreactivas, ligeramente midriáticas, sin compromiso de los pares craneales, con taquicardia sinusal en la auscultación cardiaca, en tanto que los pulmones, el abdomen y las vías genitourinarias estaban normales. En el borde externo del tendón de Aquiles, se apreciaron dos lesiones puntiformes a 6,1 mm una de la otra, con ligero eritema perilesional (figura 3A), hiperestesia en la cara interna de la pierna y el muslo, pero sin compromiso motor.

Fotografías: Gloria Esperanza Condiza-Benavides (A, D), Jhoan Manuel Ceballos-Sánchez (B, C, E, F)

A los 20 minutos, el dolor era intenso y presentó ligera dificultad respiratoria. El médico decidió aplicar dos viales de suero antiofídico polivalente liofilizado de laboratorios Probiol^®^, lote AP 009 I 19-V, a las 18:45 horas. En ese momento la tensión arterial era de 195/150 mm Hg y presentaba una ligera taquicardia. Se ordenó un inhibidor de la enzima convertidora de angiotensina (IECA) (no especificado en la historia clínica) por vía sublingual, con lo que se logró revertir el episodio hipertensivo, así como 2 g de dipirona por vía intravenosa que no tuvieron efecto sobre el dolor. La herida se lavó con solución salina al 0,9 % y jabón quirúrgico.

Se trasladó a la paciente a un hospital de nivel II en Sogamoso, Boyacá, a donde llegó a las tres horas de viaje con signos vitales estables (SO_2_: 94 %), y sin compromiso motor ni de pares craneales. El dolor intenso persistía en la región talar lesionada, y se irradiaba a toda la extremidad y la región lumbar derecha. En ese momento, el pulso periférico de la paciente era normal, el llenado capilar era de 2”, presentaba una ligera hiporreflexia patelar derecha ++ e izquierda ++++. Se decidió administrar tratamiento de gastroprotección con 50 mg de ranitidina por vía intravenosa cada 12 horas y 10 mg de metoclopramida por vía intravenosa cada 12 horas; se continuó la dipirona en dosis de 2 g, y se inició la administración intravenosa de 5.000.000 UI de penicilina cristalina cada 4 horas y de 200 mg de hidrocortisona en una sola dosis.

Un médico anotó en su historia clínica en urgencias: “…se obtiene imagen fotográfica del animal cabeza redonda, anillos pares, identificada como coral no venenosa, se inicia hidratación..*.*”. Posteriormente, se suspendió la dipirona, se le administró 1 g de acetaminofén por vía oral cada 6 horas, y se le ordenaron exámenes paraclínicos y hospitalización (cuadro 1).

**Cuadro 1:** Resultados de los exámenes paraclínicos en el Hospital Regional de Sogamoso (Boyacá) de nivel II

Estudio	Resultado
Hemograma	Leucocitos: 14,82 x 10^3^/mm^3^; neutrófilos: 90 %; linfocitos: 10 %; hemoglobina:14,6 g/dl; hematocrito: 43,6 %; plaquetas: 283 x 10^3^/mm^3^
Enzimas musculares	Creatina cinasa total: 105 U/L
Perfil metabólico	Glucemia: 88 md/dl
Función renal	Creatinina sérica: 0,99 mg/ml, Nitrógeno ureico en sangre: 10,05 mg/dl
Proteína C reactiva	0 mg/L
Electrolitos	Sodio: 139 mmol/L; potasio: 3,5 mmol/L

A las 18 horas del accidente, la paciente refiere odinofagia moderada y disgeusia: “todo lo que como, me sabe a ají”. La saturación de oxígeno era de 94 % y el dolor intenso persistía. Otro médico anotó en su historia: “se confirma especie de animal por fotografía, coral no venenosa”. Entonces, se reinició la dipirona, se suspendió la penicilina y se inició la administración de cefazolina. Se consideró el egreso hospitalario.

A las 25 horas el dolor era muy intenso y los profesionales de enfermería decidieron aplicarle 75 mg de diclofenaco por vía intravenosa en dosis única “frente a insistencia de la paciente”. Los familiares solicitaron entonces su traslado a un centro de nivel III; ingresó a la Clínica Los Andes en Tunja (Boyacá) después de 48 horas del evento y allí se aceptó el diagnóstico de accidente micrúrico por *M. ortoni*. 

La paciente presentó ligera facies de Rosenfeld con leve ptosis palpebral bilateral por paresia de la rama superior del III par craneal (figura 3B y 3C) y bradilalia con parestesia en la lengua y ligera disfagia por compromiso del IX par (glosofaríngeo) sin que progresara a los pares X (vago), XI (accesorio) y XII (hipogloso). Se ordenaron nuevos exámenes paraclínicos (cuadro 2). A pesar del manejo médico, la paciente continuó presentando hiperalgesia e hiperestesia intensas en el área de la mordedura en el miembro inferior derecho, en el tendón de Aquiles y el talón (L_5_), en el nervio sural (S_1_) en el músculo cutáneo (L_2_ a L_4_), siguiendo un curso ascendente ipsilateral hacia la región lumbosacra por estimulación de las raíces sensitivas L_1_ a S_5_, hiperestesia de T_1_ a T_12_, del hombro (nervio axilar, ramas C_5_ y C_6_), de la región anterolateral del brazo y el antebrazo (nervio radial, intercostobraquial y braquial mediano, ramas C_5_ a T_1_), parestesias en el dedo gordo derecho y en los dedos de la mano derecha (C_6_, C_7_, C_8_) ^11^.

**Cuadro 2:** Resultados de los exámenes paraclínicos en la Clínica Los Andes de Tunja (Boyacá) de nivel III

Estudio	Resultado
Hemograma	Leucocitos: 9,9 x 10^3^/mm^3^; hemoglobina: 14,2 g/dl; plaquetas: 271 x 10^3^/mm^3^
Enzimas musculares	Creatina cinasa total: 83 U/L; CK (MB): 7 U/L
Función hepática	Aspartato aminotransferasa: 15,3 UK/L; alanina- aminotransferasa: 12,8 UK/L; bilirrubina total: 0,47 mg/dl; b. directa: 0,20 mg/dl; b. indirecta: 0,27 mg/dl
Función renal	Creatinina sérica: 0,87 mg/ml; Nitrógeno ureico en sangre: 6 mg/dl
Reactante para daño tisular	Lactato deshidrogenasa: 271 U/L
Reactante para trombosis venosa profunda	Dímero D: 0,56 μg/ml
Tiempos de coagulación	Tiempo de protrombina: 12,5”/12,2”; tiempo parcial de tromboplastina: 26,6”/26,3”; INR (*International* *Normalized Ratio*): 1,13”
Ecografía abdominal total	Hallazgos normales
Ecografía Doppler venosa de miembro inferior derecho	Edemas de tejidos blandos, negativo para trombosis venosa profunda
Radiografía de tórax	Normal
Electromiografía de las cuatro extremidades	Ver descripción en el texto

Dada la persistencia del intenso dolor, el Servicio de Medicina Interna ordenó estudios con ecografía Doppler venosa y dímero D para descartar trombosis venosa profunda, los cuales fueron normales. Se le practicó una electromiografía de las cuatro extremidades, la cual mostró hallazgos de “neuropatía axonal motora y axonal-mielínica sensitiva en medianos y mielínica motora-sensitiva de tibial posterior derecho de carácter leve a moderado”. Estos hallazgos se consideraron secundarios a la neurotoxicidad del veneno de *M. ortoni*, por lo que se inició tratamiento con pregabalina y terapia física, con lo que se logró una leve disminución de los síntomas.

Al séptimo día de hospitalización, la paciente presentó exantema en pies, muslos y cara, acompañado de prurito intenso y disnea leve (figura 3D), se diagnosticó, entonces, una enfermedad del suero secundaria al uso del antiveneno polivalente de origen equino y se trató con 100 mg de hidrocortisona por vía intravenosa cada 8 horas durante tres días y 50 mg de difenhidramina por vía oral cada 8 horas durante cinco días, lo que permitió la mejoría del cuadro clínico. Dada la adecuada evolución clínica y la desaparición de las manifestaciones neurológicas (figura 3E y 3F), se ordenó el alta al noveno día, continuándose la terapia física y la pregabalina.

## Caso 2

Se trata de una mujer de 19 años mordida por una serpiente *M. ortoni* (figura 2B) cuando la pisó accidentalmente en la cocina de su casa en el casco urbano de Cartagena del Chairá, Caquetá (Colombia), situada a 350 msnm en la margen derecha del río Caguán, en la Amazonía, el día 30 de noviembre de 2020 a las 18:30 horas. El animal estaba debajo de un plástico oscuro. Existen dos lagos artificiales cerca de la casa de la paciente y, la víspera del accidente, el poblado se había inundado por el desbordamiento del río Caguán debido al intenso invierno.

**Figura 4:**
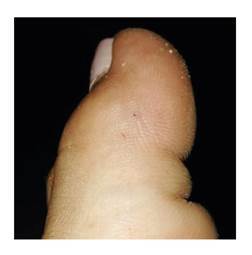
Ofidismo micrúrico por *M. ortoni* (Cartagena del Chairá). Mordedura en el dedo gordo del pie izquierdo

La paciente estaba descalza y fue mordida en la zona distal del borde externo del primer dedo del pie izquierdo (figura 4). Presentó dolor intenso y continuo, irradiado al pie y la pierna hasta la rodilla, con sensación de quemadura, por lo que acudió al centro de nivel I de atención, donde le administraron hidratación parenteral. En el examen físico, solo se encontraron las pequeñas marcas de los colmillos sin ningún otro signo, pero fue remitida al nivel II de atención en Florencia, Caquetá, por el intenso dolor que no cedió con la aplicación de tramadol intravenoso. A las dos horas, el dolor era intermitente y poco a poco fue cediendo hasta desaparecer aproximadamente tres horas después. Según los médicos, la serpiente no era una *Micrurus* sino una falsa coral.

Fotografía: Fiorella Escobar-Holguín

En el examen físico, se encontró tensión arterial de 120/80 mm Hg, frecuencia cardíaca de 75 por minuto, frecuencia respiratoria de 20 por minuto y saturación de oxígeno de 95 %. No había signos de neurotoxicidad motora ni alteraciones de los pares craneales; el resultado del resto de las evaluaciones fue negativo. Se le tomaron exámenes paraclínicos a las 18 horas de evolución (cuadro 3). El uroanálisis evidenció contaminación por menstruación.

**Cuadro 3:** Resultados de los exámenes paraclínicos en la Clínica Medilaser S. A. de Florencia (Caquetá) (nivel II)

Estudio	Resultado
Hemograma	Leucocitos: 8,5 x 10^3^/mm^3^; hemoglobina: 13,5 g/dl; plaquetas: 278 x 10^3^/mm^3^
Electrolitos	Potasio sérico: 3,64 mmol/L Sodio sérico: 141,6 mmol/L
Proteína C reactiva	3,5 mg/L
Tiempos de coagulación	Tiempo de protrombina: 15,8”/13,6”; tiempo parcial de tromboplastina: 31,7”/29,3”; INR (*International Normalized Ratio*): 1,17”
*International Normalized Ratio* (INR)	1,17”

Se trató el dolor con 500 mg de acetaminofén cada 6 horas y por vía intravenosa, 1 g de dipirona cada 8 horas y 25 mg de tramadol cada 6 horas; además, 3 g por vía intravenosa de ampicilina-sulbactam cada 8 horas y 500 ml de lactato de Ringer (70 ml/hora). Se ordenó su salida y la continuación del tratamiento por vía oral con 1 g de acetaminofén cada 8 horas por 7 días, 500 mg de cefalexina cada 8 horas por 7 días y 250 mg de naproxeno cada 8 horas por 7 días.

## Caso 3

Este caso llegó a la atención de los autores porque el herpetólogo William W. Lamar de la *University of Texas at Tyler Graduate School* estableció el enlace entre el paciente y el doctor Santiago Ayerbe, buscando orientación, ya que había ocurrido en una región latinoamericana. Se trataba de un hombre de 44 años, mordido el 27 de octubre de 2010 a las 15:50 horas en la estación biológica “Los Amigos”, Madre de Dios, en la Amazonía del Perú, por una *M. hemprichii*, denominada “coral lombricera occidental” ^5^^,^^12^ (figura 2C), en el borde interno de la zona distal del pulgar derecho mientras le tomaba fotografías. Ambos colmillos penetraron la piel. La distancia entre los colmillos se estimó entre 6 y 7 mm.

A continuación, se describe la evolución del cuadro clínico según los datos suministrados por el propio paciente, quien los tenía anotados, pues no le entregaron la epicrisis. Las cifras corresponden al tiempo en minutos y horas después de la mordedura.

“+:10’ Inicio del dolor y ligero edema en el pulgar derecho

+:45’ Entumecimiento y dolor moderado en antebrazo derecho y linfadenitis regional durante 45’

+3 h 10’ hipoestesia moderada y entumecimiento facial similar al efecto producido por anestesia local con novocaína. El síntoma duró 45.’

+4 h El dolor en el dedo, que había sido intermitente, ahora se torna severo (sic) en todo el dedo y en oleadas por cerca de 30.’

+7 h El dolor ha disminuido notablemente. A las 06:00 horas del 28 de junio, no hay dolor ni edema”.

Aproximadamente a las 12:00 horas del 29 de junio, el paciente refiere dolor difuso en todo el cuerpo similar al de un resfriado común y palpa una adenopatía dolorosa en la parte lateral derecha del cuello. Este cuadro clínico duró ocho horas. No se presentaron más síntomas.

El paciente fue evacuado de la estación biológica hacia un hospital en Puerto Maldonado, cerca de la medianoche del día del evento; la doctora que lo atendió se negó a creer que había sido mordido por una *Micrurus*, pues tenía otro concepto sobre el patrón de estas serpientes, desconocía el agente causal, *M*. *hemprichii*, y la evolución clínica del paciente tampoco encajaba en el cuadro clínico clásico del ofidismo micrúrico. Por otra parte, la médica desestimó la identificación positiva de la serpiente, hecha por un herpetólogo de reconocida trayectoria internacional. Esta situación generó desconcierto en el paciente. Le tomaron una muestra de sangre y le aplicaron tetanol. Fue enviado a un hotel donde lo examinaron cada 30 minutos por si empeoraba, hasta la mañana del 30 de junio cuando le dieron de alta.

### Consideraciones éticas

Se garantizó la confidencialidad de la información según lo estipulado en la Ley 1273 del 2009 y la Ley 1266 del mismo año. Los pacientes autorizaron la publicación de los casos y el uso de fotografías.

## Discusión

En Colombia y Perú, el accidente ofídico se considera un problema de salud pública de notificación obligatoria, ya que la diversidad de ecosistemas y clima de ambos países permiten albergar muchas especies de serpientes, entre las cuales se encuentran dos familias de serpientes venenosas: Viperidae y Elapidae. Las familias Colubridae y Dipsadidae tienen algunas serpientes con dentición opistoglifa y son potencialmente venenosas, aunque no letales ^5^.

En un estudio de los venenos de ocho corales amazónicas y una de norteamérica, se encontró que el veneno de *M. hemprichii* ocupa el sexto lugar en letalidad, con una dosis letal 50 (DL_50_) de 2,35 mg/kg (47 μg/ratón de 18-22 g), actividad específica de la fosfolipasa A_2_ de 24 nm/minuto/μg de veneno asociada a neurotoxicidad y actividad proteolítica. En cambio, ocupa el tercer lugar en cuanto a actividad de la hialuronidasa, casi igual a la observada en *M. lemniscatus*^12^. En otro estudio, la DL_50_ varío entre 1,41 y 3,4 mg/kg por vía intraperitoneal e intramuscular, respectivamente (Ẋ=2,4 mg/kg), ocupando el tercer lugar después de *M. surinamensis* y *M. lemniscatus*^13^. El antiveneno anticoral fabricado por el Instituto Butantán (Brasil) ha demostrado una buena capacidad de neutralización moderada del efecto de la fosfolipasa A_2_, la actividad proteolítica y la hialuronidasa del veneno de *M. hemprichii*^12^.

El cuadro clínico de los pacientes presentados, no progresó a parálisis flácida respiratoria y periférica, quizá debido a que el veneno de *M. ortoni* es uno de los menos potentes, como se demostró en el estudio comparativo con ocho venenos de corales amazónicas ^6^. Por ello, se deduce que su contenido de α-neurotoxinas es bajo, tiene predominio de acción en las fibras neurosensoriales, y su diseminación se produce por una vía diferente, pues esta no fue global sino ascendente e ipsilateral a la lesión, sin manifestaciones en el hemicuerpo contralateral. En este estudio, no se comprobó el efecto miotóxico descrito para *M. ortoni*^6^. Asimismo, no se han reportado las alteraciones de la coagulación descritas con las *Micrurus* spp. de norteamérica y Venezuela ^14^*.*

El veneno de *Micrurus* tiene una actividad neurotóxica potente, aunque la actividad miotóxica o hemolítica de algunas especies puede ser escasa o estar ausente. Las neurotoxinas interfieren en la transmisión colinérgica en el sistema nervioso periférico y en la unión neuromuscular. Se distinguen tres tipos de neurotoxinas: α-neurotoxinas, κ-neurotoxinas y muscarinotoxinas. Las α-neurotoxinas son tridigitotoxinas que inhiben la acetilcolina y bloquean la unión postsináptica neuromuscular 15 a 20 veces más que la dextrotubocurarina en los receptores α-1n-ACh, y son poco reversibles con la neostigmina ^15^^,^^16^. Actualmente, se han identificado nuevas neurotoxinas del tipo de las tridigitotoxinas en venenos de *M. mipartitus* y *M. dumerilii*, cuya actividad no ha sido letal en ratones, y que marcan diferencias filogenéticas importantes entre estas especies ^17^^,^^18^. Sin embargo, no hay estudios sobre la proteómica del veneno de *M. hemprichii* y *M. ortoni* que pudiesen explicar la poca toxicidad del veneno de *M. hemprichii* y *M. ortoni*, ni su efecto predominantemente sensitivo.

Los accidentes ofídicos por serpientes del género *Micrurus* se caracterizan por la ausencia de efectos patológicos locales y en la coagulación, y por el predominio de la actividad de neurotoxinas que bloquean las uniones neuromusculares a nivel presináptico y postsináptico ^14^. Se ha descrito miotoxicidad por dos especies de corales amazónicas, incluído un caso de *M. hemprichii*^12^, así como efectos miotóxicos experimentales en 11 especies de *Micrurus*^19^ y alteraciones de la coagulación sanguínea por dos especies de *Micrurus* de norteamérica y una de sudamérica ^14^, pero dicha actividad no es común a la gran mayoría de los venenos de este género de serpientes.

El cuadro clínico clásico producido por la mordedura de *Micrurus* se caracteriza por parálisis de los pares craneales, con paro respiratorio asociado a parálisis flácida de los músculos intercostales y del diafragma, así como de los músculos de todo el cuerpo. No se presentan cambios inflamatorios locales y puede haber dolor leve, parestesia local (descrita por los pacientes como “hormigueo” o “adormecimiento”) y sensación de picazón, ardor, quemadura o prurito, según lo que ha podido establecerse en los estudios con base en la experiencia de atención de casos ^3^. Como se evidenció en los casos 1 y 3, estos síntomas son pasajeros.

Generalmente, los síntomas por envenenamiento aparecen alrededor de una hora después de la mordedura, pero si no hay signos a las seis horas, lo más seguro es que se trate de una mordedura en seco, lo cual es relativamente frecuente cuando la serpiente muerde sobre una superficie plana, como el dorso de la mano o el pie, pues raras veces logra abrir sus fauces más de 45 grados; en este caso no se aplica antiveneno ^5^.

En los casos 1 y 3, debió utilizarse el antiveneno anticoral del Instituto Butantán (São Paulo, Brasil), que ha demostrado ser parcialmente efectivo por tener inmunidad cruzada inmunoquímica con el veneno de *M. hemprichii* en dosis de cinco viales, con lo cual solo se presenta síndrome miasténico leve y dolor sin progresión a parálisis respiratoria ^20^. En el segundo caso, no se requería antiveneno.

En el primer caso del presente reporte, el paciente cursó con la sintomatología típica del ofidismo micrúrico leve en cuanto al compromiso motor ^5^^,^^9^, en tanto que predominó el dolor intenso (hiperalgesia e hiperestesia), lo que solo se observa en 9,3 % de los casos ^20^. Con el transcurrir del tiempo, hubo hipoestesia, es decir que al comienzo solo estaba actuando la toxina en las terminales neurosensitivas. En este caso, se presentó una emergencia hipertensiva grave para la edad de la paciente después de la transfusión del antiveneno, lo que no se ha descrito en otros casos de ofidismo micrúrico y pudo revertirse con un inhibidor de la ECA administrado por vía sublingual y no especificado. Creemos que la emergencia hipertensiva se debió a la liberación de catecolaminas por el estrés y la angustia de la paciente.

Llama la atención que en los casos 1 y 3 el dolor se presentó en el hemicuerpo ipsilateral a la zona de la mordedura, es decir que la neurotoxina responsable de la hiperalgesia se diseminó al parecer por vía linfática subcutánea. Este cuadro clínico se debe parcialmente al compromiso motor evidenciado en la electromiografía, en la cual se encontró neuropatía axonal motora y axonal mielínica sensitiva en medianos, y mielínica motora sensitiva del tibial posterior derecho de carácter leve a moderado. Clínicamente, solo se observó una discreta ptosis palpebral en el primer caso, que no progresó a los otros pares craneales ni a los nervios intercostales o periféricos. El hemograma registró una ligera leucocitosis en el primer caso y fue normal en el segundo; la función renal se conservó, los tiempos de coagulación, la CPK total, la CPK MB, y la función hepática fueron normales, así como la natremia y los niveles de potasio, es decir, no hubo evidencia de hemotoxicidad ni miotoxicidad (cuadros 1, 2  y 3), aunque hay que anotar que los resultados de los exámenes paraclínicos se desconocen en el tercer caso.

En los niveles I y II de atención, se constató el desconocimiento por parte del cuerpo médico del agente causal (*M. hemprichii* y *M. ortoni*), su veneno y de los grados de envenenamiento en los tres casos, pues se administró un antiveneno que no estaba indicado para elápidos (corales) sino para tres géneros de vipéridos (víboras), cuyos venenos son completamente diferentes y no presentan ninguna reactividad cruzada con el veneno micrúrico. Se prescribió pregabalina como analgésico, lo que demostró alguna efectividad, al parecer, porque este medicamento anticonvulsivo y analgésico actúa bloqueando el calcio en la unión presináptica y las α-neurotoxinas del veneno micrúrico que producen un bloqueo nicotínico de los receptores ionotrópicos de la acetilcolina actúan en la unión postsináptica ^13^^,^^14^. Sin embargo, este medicamento potencia el bloqueo neuromuscular, por lo cual no fue tan efectivo contra el dolor. Aunque el dolor causado por el ofidismo botrópico se trata con dipirona, un inhibidor de la ciclooxigenasa precursora de las prostaglandinas, no fue efectivo en este caso, como tampoco el diclofenaco, pues no había un proceso inflamatorio, como lo confirmó la ausencia de proteína C reactiva.

Consideramos que en los casos 1 y 3 debió emplearse tramadol pues, a pesar de actuar como un opioide, tiene menor riesgo de producir depresión respiratoria y es un potente analgésico, como se vio en el segundo caso. Para reducir el efecto colateral de las globulinas de origen equino (cefalea, escalofríos, hiperemia conjuntival, etc.), según la experiencia, se recomienda aplicar un antihistamínico (hidroxicina, clemastina o difenhidramina) 15 minutos antes de aplicar el antiveneno. El uso de esteroides solo se recomienda para tratar la enfermedad del suero ^5^.

## Conclusiones

El cuadro clínico de los tres pacientes no progresó a parálisis flácida respiratoria o periférica, tal vez debido al escaso efecto letal del veneno de *M. ortoni* demostrado en los estudios de corales amazónicas. Se deduce que el contenido de α-neurotoxinas es bajo en esta especie y actúa predominantemente en las fibras neurosensitivas; además, su diseminación se dio por una vía diferente, pues esta no fue global sino ascendente e ipsilateral a la lesión, sin manifestaciones en el hemicuerpo contralateral. No se comprobó el efecto miotóxico de *M. ortoni*, ni las alteraciones de la coagulación producidas por *Micrurus* spp. de norteamérica y Venezuela.

El manejo, aunque oportuno, presentó fallas en el uso del antiveneno; además, en los tres casos se evidenció el desconocimiento de la especie causal y su sintomatología por parte del cuerpo médico. Por ello, debe insistirse en la creación de la cátedra de toxinología en las universidades, ya que la biodiversidad de especies animales y vegetales es grande en el neotrópico y persiste el desconocimiento de una condición desatendida que afecta a la población de bajos recursos, siempre vulnerable al efecto de los venenos de origen natural, por lo que es necesario consultar con centros especializados o expertos que ayuden en la correcta identificación.

La neurografía motora y sensitiva y la electromiografía demuestran ser de gran utilidad en el tratamiento de los pacientes con mordeduras de especies micrúricas, pues permiten hacer una correlación apropiada con la clínica, revelando el grado y el lugar de la afección motora y sensitiva.

En este estudio, se confirma una vez más que esta clase de ofidismo se puede tratar adecuadamente mediante el tratamiento sintomático. El antiveneno antimicrúrico apropiado solo sirve si se aplica en las primeras tres o cuatro horas del accidente; después, ya es inefectivo, pues las neurotoxinas de bajo peso molecular se fijan velozmente en las placas neuromotoras y sensitivas, donde es metabolizado sin dejar secuelas graves.

Se sigue observando la aparición de la enfermedad del suero y de reacciones colaterales importantes con el uso de algunos antivenenos de origen equino ^21^. Al no recibir el antiveneno específico para *Micrurus* spp., los tres pacientes deben haber desarrollado inmunidad natural contra el veneno de *M. ortoni* y *M. hemprichii*. Sería interesante hacer un estudio inmunológico con ellos para confirmar esta hipótesis.
